# Supplementary material for: Humoral Immune Response in Immunized Sheep with Bovine Coronavirus Glycoproteins Delivered via an Adenoviral Vector
Source: Pathogens. 2024 Jun 21;13(7):523. doi: 10.3390/pathogens13070523 (PMC11280461; doi:10.3390/pathogens13070523)
Supplement: Supplementary file 1 [file pathogens-13-00523-s001.zip › pathogens-3039664-supplementary.pdf]

Supplementary Figure S1: Sequence of Glycoprotein M, M-HA, M-HA codon usage adapted and traslated ORF.

Glycoprotein M
ATGAGTAGTGTAACTACACCAGCACCAGTTTACACCTGGACTGCTGATGA
AGCTATTAAATTCCTAAAGGAATGGAACTTTTCTTTGGGTATTATACTACT
TTTTATTACAGTCATATTGCAATTTGGATACACAAGTCGCAGTATGTTTGT
TTATGTTATTAAGATGATCCTTTTGTGGCTTATGTGGCCCCTTACTATCAT
CTTAACTATTTTTAATTGCGTGTATGCTTTGAATAATGTGTATCTTGGCTTT
TCTATAGTTTTCACTATAGTGGCCATTATCATGTGGATTGTGTATTTTGTG
AATAGTATCAGGTTGTTTATTAGAACTGGAAGTTGGTGGAGTTTCAACCC
AGAAACAAACAACTTGATGTGTATAGATATGAAAGGAAGGATGTATGTT
AGGCCTATAATTGAGGACTACCACACCCTTACGGTCACAATAATACGTGG
TCATCTTTACATGCAAGGTATAAAACTAGGTACTGGCTATTCTTTGTCTGA
TTTGCCAGCTTATGTGACTGTTGCTAAGGTATCACACCTGCTCACGTATAA
GCGTGGTTTTCTTGACAAGATAGGCGATACTAGTGGTTTTGCTGTTTATGT
TAAGTCCAAAGTCGGTAATTACCGACTGCCATCAACCCAAAAGGGTTCTG
GCATGGACACCGCATTGTTGAGAAATAATATCTAA

Glycoprotein M-HA
ATGAGTAGTGTAACTACACCAGCACCAGTTTACACCTGGACTGCTGATGA
AGCTATTAAATTCCTAAAGGAATGGAACTTTTCTTTGGGTATTATACTACT
TTTTATTACAGTCATATTGCAATTTGGATACACAAGTCGCAGTATGTTTGT
TTATGTTATTAAGATGATCCTTTTGTGGCTTATGTGGCCCCTTACTATCAT
CTTAACTATTTTTAATTGCGTGTATGCTTTGAATAATGTGTATCTTGGCTTT
TCTATAGTTTTCACTATAGTGGCCATTATCATGTGGATTGTGTATTTTGTG
AATAGTATCAGGTTGTTTATTAGAACTGGAAGTTGGTGGAGTTTCAACCC
AGAAACAAACAACTTGATGTGTATAGATATGAAAGGAAGGATGTATGTT
AGGCCTATAATTGAGGACTACCACACCCTTACGGTCACAATAATACGTGG
TCATCTTTACATGCAAGGTATAAAACTAGGTACTGGCTATTCTTTGTCTGA
TTTGCCAGCTTATGTGACTGTTGCTAAGGTATCACACCTGCTCACGTATAA
GCGTGGTTTTCTTGACAAGATAGGCGATACTAGTGGTTTTGCTGTTTATGT
TAAGTCCAAAGTCGGTAATTACCGACTGCCATCAACCCAAAAGGGTTCTG
GCATGGACACCGCATTGTTGAGAAATAATATCTACCCCTACGACGTGCCC
GATTACGCCTAA

Glycoprotein M-HA codon usage adapted
ATGAGCAGCGTGACCACCCCCGCCCCCGTGTACACCTGGACCGCCGACGA
GGCCATCAAGTTCCTGAAGGAGTGGAACTTCAGCCTGGGCATCATCCTGC
TGTTCATCACCGTGATCCTGCAGTTCGGCTACACCAGCCGCAGCATGTTC
GTGTACGTGATCAAGATGATCCTGCTGTGGCTGATGTGGCCCCTGACCAT
CATCCTGACCATCTTCAACTGCGTGTACGCCCTGAACAACGTGTACCTGG
GCTTCAGCATCGTGTTCACCATCGTGGCCATCATCATGTGGATCGTGTACT
TCGTGAACAGCATCCGCCTGTTCATCCGCACCGGCAGCTGGTGGAGCTTC
AACCCCGAGACCAACAACCTGATGTGCATCGACATGAAGGGCCGCATGT
ACGTGCGCCCCATCATCGAGGACTACCACACCCTGACCGTGACCATCATC
CGCGGCCACCTGTACATGCAGGGCATCAAGCTGGGCACCGGCTACAGCCT
GAGCGACCTGCCCGCCTACGTGACCGTGGCCAAGGTGAGCCACCTGCTGA
CCTACAAGCGCGGCTTCCTGGACAAGATCGGCGACACCAGCGGCTTCGCC
GTGTACGTGAAGAGCAAGGTGGGCAACTACCGCCTGCCCAGCACCCAGA
AGGGCAGCGGCATGGACACCGCCCTGCTGCGCAACAACATCTACCCCTAC
GACGTGCCCGACTACGCCTAA

Translated ORF
MSSVTTPAPVYTWTADEAIKFLKEWNFSLGIILLFITVILQFGYTSRSMFVYVI
KMILLWLMWPLTIILTIFNCVYALNNVYLGFSIVFTIVAIIMWIVYFVNSIRLFI
RTGSWWSFNPETNNLMCIDMKGRMYVRPIIEDYHTLTVTIIRGHLYMQGIKL
GTGYSLSDLPAYVTVAKVSHLLTYKRGFLDKIGDTSGFAVYVKSKVGNYRL
PSTQKGSGMDTALLRNNIYPYDVPDYA-

Supplementary Figure S2: Sequence of Glycoprotein S, S-ΔRS-HA S-ΔRS-HA codon usage adapted and traslated ORF

Glycoprotein S

ATGTTTTTGATACTTTTAATTTCCTTACCTACGGCTTTTGCTGTTATAGGAG
ATTTAAAGTGTACTACAGTTTCCATTAATGATGTTGACACTGGTGTTCCTT
CTATTAGCACTGATACTGTTGATGTTACTAATGGTTTAGGTACTTACTATG
TTTTAGATCGTGTGTATTTAAATACTACCTTGTTGCTTAATGGTTATTATCC
TACTTCAGGTTCTACATATCGTAATATGGCACTGAAGGGAACTTTACTATT
GAGCACACTATGGTTTAAACCACCTTTTCTTTCTGATTTTACTAATGGTAT
TTTTGCTAAGGTCAAAAACACCAAGGTTAATAAAAAGGGTGTAATGTATA
GTGAGTTTCCTGCTATAACTATAGGTAGTACTTTTGTAAATACTTCCTATA
GTGTGGTAGTACAACCATATACTACCAATTTTGATAACAAAATACAAGGT
CTCTTAGAGGTCTCTGTTTGCCAGTATACTATGTGTGAGTACCCAAACACG
ATTTGTAATCCTAATCTGGGTAATAAACGCGTAGAACTATGGCATTGGGA
TACAGGTGTTGTCTCCTGTTTATATAAGCGTAATTTCACTTATGATGTGAA
TGCTGATTATTTGTATTTCCATTTTTATCAAGAAGGTGGTACTTTTTATGC
ATATTTTACAGACACTGGTGTTGTTACTAAGTTTCTGTTTAATGTATATTT
AGGCACGGTGCTTTCACACTATTATGTCATGCCTTTGACTTGTAATAGTGC
TTTGAAGTTAGAATATTGGGTTACACCTCTCACTTCTAAACAATATTTACT
CGCTTTCAATCAAGATGGTGTTATTTTTAATGCTGTTGATTGTAAGAGTGA
TTTTATGAGTGAGATTAAGTGTAAAACATTATCTATAGCACCATTGACTG
GTGTTTATGAATTAAACGGTTACACTGTTCAGCCAATTGCAGATGTTTACC
GACGTATACCTACTCTTCCCGATTGTAATATAGAGGCTTGGCTTAATGATA
AGTCGGTGCCCTCTCCATTAAATTGGGAACGTAAGACCTTTTCAAATTGT
AATTTTAATATGAGCAGCCTGATGTCTTTTATTCAGGCAGACTCATTTATT
TGTAATAATATTGATGCTGCTAAGATATATGGTATGTGTTTTTCCAGCATA
ACTATAGATAAGTTTGCTATACCCAATGGCAGAAAGGTTGACCTACAATT
GGGCAATTTGGGTTATTTGCAGTCTTTTAATTATAGAATTGATACTACTGC
TACAAGTTGTCAGTTGTATTATAATTTACCTGCTGCTAATGTTTCTGTTAG
CAGGTTTAATCCTTCTACTTGGAATAGGAGATTTGGTTTTACAGAACAATC
TGTTTTTAAGCCTCAACCTGCAGGTGTCTTTACTGATCATGATGTTGTTTA
TGCACAACATTGTTTTAAAGCTCCCACAAATTTCTGTCCGTGTAAATTGGA
TGGGTCTTTGTGTGTAGGTAGTGGTTCTGGTATAGATGCTGGTTATAAAAC
TAGTGGTATAGGCACTTGTCCTGCAGGTACTAATTATTTAACTTGTTATAA
TGCTGCCCAATGTGACTGTATGTGCACCCCAGACCCCATTACATCTAAAG
CTACAGGGCCTTACAAGTGCCCCCAAACTAAATATTTAGTTGGCATAGGT
GAGCACTGTTCAGGTCTTGCTATTAAAAGTGATCATTGTGGAGGTAATCC
ATGTACTTGCCAACCACAAGCATTTTTGGGTTGGTCTGTTGACTCTTGTTT
ACAAGGGGATAGGTGTAATATTTTTGCTAATTTTATTTTGCATGGTGTTAA
TAGTGGTACTACTTGTTCTACTGATTTACAAAAATCAAACACAGACATAA
TTCTTGGTGTTTGTGTTAATTATGATCTTTATGGTATTACTGGCCAAGGTA
TTTTTGTTGAGGTTAATGCGACTTATTATAATAGTTGGCAGAACCTTTTGT
ATGATTCTAATGGTAATCTCTATGGTTTTAGAGACTACTTAACAAACAGA

ACTTTCATGATTCGTAGTTGCTATAGCGGTCGTGTTTCAGCGGCCTTTCAT
GCTAACTCTTCCGAACCAGCATTGCTATTTCGGAATATTAAATGCAACTA
CGTTTTTAATAATACTCTTTCACGACAGCTGCAACCTATTAACTATTTTGA
TAGCTATCTTGGTTGTGTTGTCAATGCTGATAATAGTACTTCTAGTGTTGT
TCAAACATGTGATCTCACAGTAGGTAGTGGTTACTGTGTGGATTACTCTA
CAAAAAGACGAAGTCGTGGAGCGATTACCACTGGTTATCGGTTTACTAAT
TTTGAGCCATTTACTGTTAAGTCAGTAAATGATAGTTTAGAACCTGTAGGT
GGTTTGTATGAAATTCAAATACCTTCAGATTTTACTATAGGTAACATGGA
GGAGTTTATTCAAACAAGCTCTCCTAAAGTTACTATTGATTGTTCTACTTT
TGTCTGTGGTGATTATGCAGCATGTAAATCACAGTTGGTTGAATATGGTA
GCTTTTGTGATAATATTAATGCTATACTCACAGAAGTAAATGAACTACTT
GACACTACACAGTTGCAAGTAGCTAATAGTTTAATGAATGGTGTCACCCT
TAGCACTAAGCTTAAAGATGGCGTTAAGTTCAATGTAGACGACATCAATT
TTTCCCCTGTATTAGGTTGTGTAGGAAGCGATTGTAATAGAGTTTCCAGTA
GATCTGCTATAGAGGATTTACTTTTTTCTAAAGTAAAGTTAGCTGATGTCG
GTTTTGTTGAGGCTTATAATAATTGTACTGGAGGTGCCGAAATTAGGGAC
CTCATTTGTGTGCAAAGTTATAATGGTATTAAGGTCTTGCCTCCACTGCTC
TCAGAAAATCAGATCAGTGGATATACTTTAGCTGCGACCTCTGCTAGTTT
GTTTCCCCCTTGGTCAGCAGCAGCAGGTGTACCATTTTATTTAAATGTTCA
GTATCGTATTAATGGGATTGGTGTTACCATGGATGTGCTAAGTCAAAATC
AAAAGCTTATTGCTAATGCATTTAACAATGCCCTTGGTGCTATCCAGGAA
GGGTTTGATGCTACTAATTCTGCTTTAGTCAAAATTCAAGCTGTTGTTAAT
GCAAATGCTGAAGCTCTTAATAACTTATTGCAACAACTCTCTAATAGGTTT
GGTGCTATAAGTTCTTCTTTACAAGAAATTCTATCTAGACTGGATGCTCTT
GAAGCGCAAGCTCAGATAGACAGACTTATTAATGGGCGTCTTACCGCTCT
TAATGCTTATGTTTCTCAACAGCTTAGTGACTCTACACTAGTAAAATTTAG
TGCAGCACAAGCTATGGAGAAGGTTAATGAATGTGTCAAAAGCCAATCA
TCTAGGATAAATTTTTGTGGTAATGGTAATCATATTATATCATTAGTGCAG
AATGCTCCATATGGTTTGTATTTTATCCACTTTAGCTATGTCCCTACTAAG
TATGTCACTGCGAAGGTTAGTCCTGGTCTGTGCATTGCTGGTGATAGAGG
TATAGCCCCCAAGAGTGGTTATTTTGTTAATGTAAATAATACTTGGATGTT
CACTGGTAGTGGTTACTACTACCCTGAACCTATAACTGGAAATAATGTTG
TTGTTATGAGTACCTGTGCTGTTAATTATACTAAAGCACCGGATGTAATGC
TGAACATTTCAACACCCAACCTCCCCGATTTTAAGGAAGAGTTGGACCAA
TGGTTTAAAAACCACACATCAGTGGCACCCAATTTGTCACTTGATTATAT
AAATGTTACATTCTTGGATCTACAAGATGAAATGAATAGGTTACAGGAGG
CAATAAAAGTTTTAAATCAGAGCTACATCAATCTCAAGGACATTGGTACA
TATGAGTATTATGTAAAATGGCCTTGGTATGTATGGCTTTTAATTGGCTTT
GCTGGTGTAGCTATGCTTGTTTTACTATTCTTCATATGCTGTTGCACAGGA
TGTGGGACTAGTTGTTTTAAGAAATGTGGTGGTTGTTGTGATGATTATACT
GGACACCAGGAGTTAGTAATTAAAACATCACATGACGACTAA

Glycoprotein S-ΔRS-HA
ATGTTTTTGATACTTTTAATTTCCTTACCTACGGCTTTTGCTGTTATAGGAG
ATTTAAAGTGTACTACAGTTTCCATTAATGATGTTGACACTGGTGTTCCTT
CTATTAGCACTGATACTGTTGATGTTACTAATGGTTTAGGTACTTACTATG
TTTTAGATCGTGTGTATTTAAATACTACCTTGTTGCTTAATGGTTATTATCC
TACTTCAGGTTCTACATATCGTAATATGGCACTGAAGGGAACTTTACTATT
GAGCACACTATGGTTTAAACCACCTTTTCTTTCTGATTTTACTAATGGTAT
TTTTGCTAAGGTCAAAAACACCAAGGTTAATAAAAAGGGTGTAATGTATA
GTGAGTTTCCTGCTATAACTATAGGTAGTACTTTTGTAAATACTTCCTATA
GTGTGGTAGTACAACCATATACTACCAATTTTGATAACAAAATACAAGGT
CTCTTAGAGGTCTCTGTTTGCCAGTATACTATGTGTGAGTACCCAAACACG
ATTTGTAATCCTAATCTGGGTAATAAACGCGTAGAACTATGGCATTGGGA
TACAGGTGTTGTCTCCTGTTTATATAAGCGTAATTTCACTTATGATGTGAA
TGCTGATTATTTGTATTTCCATTTTTATCAAGAAGGTGGTACTTTTTATGC
ATATTTTACAGACACTGGTGTTGTTACTAAGTTTCTGTTTAATGTATATTT
AGGCACGGTGCTTTCACACTATTATGTCATGCCTTTGACTTGTAATAGTGC
TTTGAAGTTAGAATATTGGGTTACACCTCTCACTTCTAAACAATATTTACT
CGCTTTCAATCAAGATGGTGTTATTTTTAATGCTGTTGATTGTAAGAGTGA
TTTTATGAGTGAGATTAAGTGTAAAACATTATCTATAGCACCATTGACTG
GTGTTTATGAATTAAACGGTTACACTGTTCAGCCAATTGCAGATGTTTACC
GACGTATACCTACTCTTCCCGATTGTAATATAGAGGCTTGGCTTAATGATA
AGTCGGTGCCCTCTCCATTAAATTGGGAACGTAAGACCTTTTCAAATTGT
AATTTTAATATGAGCAGCCTGATGTCTTTTATTCAGGCAGACTCATTTATT
TGTAATAATATTGATGCTGCTAAGATATATGGTATGTGTTTTTCCAGCATA
ACTATAGATAAGTTTGCTATACCCAATGGCAGAAAGGTTGACCTACAATT
GGGCAATTTGGGTTATTTGCAGTCTTTTAATTATAGAATTGATACTACTGC
TACAAGTTGTCAGTTGTATTATAATTTACCTGCTGCTAATGTTTCTGTTAG
CAGGTTTAATCCTTCTACTTGGAATAGGAGATTTGGTTTTACAGAACAATC
TGTTTTTAAGCCTCAACCTGCAGGTGTCTTTACTGATCATGATGTTGTTTA
TGCACAACATTGTTTTAAAGCTCCCACAAATTTCTGTCCGTGTAAATTGGA
TGGGTCTTTGTGTGTAGGTAGTGGTTCTGGTATAGATGCTGGTTATAAAAC
TAGTGGTATAGGCACTTGTCCTGCAGGTACTAATTATTTAACTTGTTATAA
TGCTGCCCAATGTGACTGTATGTGCACCCCAGACCCCATTACATCTAAAG
CTACAGGGCCTTACAAGTGCCCCCAAACTAAATATTTAGTTGGCATAGGT
GAGCACTGTTCAGGTCTTGCTATTAAAAGTGATCATTGTGGAGGTAATCC
ATGTACTTGCCAACCACAAGCATTTTTGGGTTGGTCTGTTGACTCTTGTTT
ACAAGGGGATAGGTGTAATATTTTTGCTAATTTTATTTTGCATGGTGTTAA
TAGTGGTACTACTTGTTCTACTGATTTACAAAAATCAAACACAGACATAA
TTCTTGGTGTTTGTGTTAATTATGATCTTTATGGTATTACTGGCCAAGGTA
TTTTTGTTGAGGTTAATGCGACTTATTATAATAGTTGGCAGAACCTTTTGT
ATGATTCTAATGGTAATCTCTATGGTTTTAGAGACTACTTAACAAACAGA
ACTTTCATGATTCGTAGTTGCTATAGCGGTCGTGTTTCAGCGGCCTTTCAT
GCTAACTCTTCCGAACCAGCATTGCTATTTCGGAATATTAAATGCAACTA
CGTTTTTAATAATACTCTTTCACGACAGCTGCAACCTATTAACTATTTGA

TAGCTATCTTGGTTGTGTTGTCAATGCTGATAATAGTACTTCTAGTGTTGT
TCAAACATGTGATCTCACAGTAGGTAGTGGTTACTGTGTGGATTACTCTA
CAAAAAGACGAAGTCGTGGAGCGATTACCACTGGTTATCGGTTTACTAAT
TTTGAGCCATTTACTGTTAAGTCAGTAAATGATAGTTTAGAACCTGTAGGT
GGTTTGTATGAAATTCAAATACCTTCAGATTTTACTATAGGTAACATGGA
GGAGTTTATTCAAACAAGCTCTCCTAAAGTTACTATTGATTGTTCTACTTT
TGTCTGTGGTGATTATGCAGCATGTAAATCACAGTTGGTTGAATATGGTA
GCTTTTGTGATAATATTAATGCTATACTCACAGAAGTAAATGAACTACTT
GACACTACACAGTTGCAAGTAGCTAATAGTTTAATGAATGGTGTCACCCT
TAGCACTAAGCTTAAAGATGGCGTTAAGTTCAATGTAGACGACATCAATT
TTTCCCCTGTATTAGGTTGTGTAGGAAGCGATTGTAATAGAGTTTCCAGTA
GATCTGCTATAGAGGATTTACTTTTTTCTAAAGTAAAGTTAGCTGATGTCG
GTTTTGTTGAGGCTTATAATAATTGTACTGGAGGTGCCGAAATTAGGGAC
CTCATTTGTGTGCAAAGTTATAATGGTATTAAGGTCTTGCCTCCACTGCTC
TCAGAAAATCAGATCAGTGGATATACTTTAGCTGCGACCTCTGCTAGTTT
GTTTCCCCCTTGGTCAGCAGCAGCAGGTGTACCATTTTATTTAAATGTTCA
GTATCGTATTAATGGGATTGGTGTTACCATGGATGTGCTAAGTCAAAATC
AAAAGCTTATTGCTAATGCATTTAACAATGCCCTTGGTGCTATCCAGGAA
GGGTTTGATGCTACTAATTCTGCTTTAGTCAAAATTCAAGCTGTTGTTAAT
GCAAATGCTGAAGCTCTTAATAACTTATTGCAACAACTCTCTAATAGGTTT
GGTGCTATAAGTTCTTCTTTACAAGAAATTCTATCTAGACTGGATGCTCTT
GAAGCGCAAGCTCAGATAGACAGACTTATTAATGGGCGTCTTACCGCTCT
TAATGCTTATGTTTCTCAACAGCTTAGTGACTCTACACTAGTAAAATTTAG
TGCAGCACAAGCTATGGAGAAGGTTAATGAATGTGTCAAAAGCCAATCA
TCTAGGATAAATTTTTGTGGTAATGGTAATCATATTATATCATTAGTGCAG
AATGCTCCATATGGTTTGTATTTTATCCACTTTAGCTATGTCCCTACTAAG
TATGTCACTGCGAAGGTTAGTCCTGGTCTGTGCATTGCTGGTGATAGAGG
TATAGCCCCCAAGAGTGGTTATTTTGTTAATGTAAATAATACTTGGATGTT
CACTGGTAGTGGTTACTACTACCCTGAACCTATAACTGGAAATAATGTTG
TTGTTATGAGTACCTGTGCTGTTAATTATACTAAAGCACCGGATGTAATGC
TGAACATTTCAACACCCAACCTCCCCGATTTTAAGGAAGAGTTGGACCAA
TGGTTTAAAAACCACACATCAGTGGCACCCAATTTGTCACTTGATTATAT
AAATGTTACATTCTTGGATCTACAAGATGAAATGAATAGGTTACAGGAGG
CAATAAAAGTTTTAAATCAGAGCTACATCAATCTCAAGGACATTGGTACA
TATGAGTATTATGTAAAATGGCCTTGGTATGTATGGCTTTTAATTGGCTTT
GCTGGTGTAGCTATGCTTGTTTTACTATTCTTCATATGCTGTTGCACAGGA
TGTGGGACTAGTTGTTTTAAGAAATGTGGTGGTTACCCCTACGACGTGCC
CGATTACGCCTAA

Glycoprotein S-ΔRS-HA codon usage adapted

ATGTTCCTGATCCTGCTGATCAGCCTGCCCACCGCCTTCGCCGTGATCGGC
GACCTGAAGTGCACCACCGTGAGCATCAACGACGTGGACACCGGCGTGC
CCAGCATCAGCACCGACACCGTGGACGTGACCAACGGCCTGGGCACCTA
CTACGTGCTGGACCGCGTGTACCTGAACACCACCCTGCTGCTGAACGGCT
ACTACCCCACCAGCGGCAGCACCTACCGCAACATGGCCCTGAAGGGCAC
CCTGCTGCTGAGCACCCTGTGGTTCAAGCCCCCCTTCCTGAGCGACTTCAC
CAACGGCATCTTCGCCAAGGTGAAGAACACCAAGGTGAACAAGAAGGGC
GTGATGTACAGCGAGTTCCCCGCCATCACCATCGGCAGCACCTTCGTGAA
CACCAGCTACAGCGTGGTGGTGCAGCCCTACACCACCAACTTCGACAACA
AGATCCAGGGCCTGCTGGAGGTGAGCGTGTGCCAGTACACCATGTGCGA
GTACCCCAACACCATCTGCAACCCCAACCTGGGCAACAAGCGCGTGGAG
CTGTGGCACTGGGACACCGGCGTGGTGAGCTGCCTGTACAAGCGCAACTT
CACCTACGACGTGAACGCCGACTACCTGTACTTCCACTTCTACCAGGAGG
GCGGCACCTTCTACGCCTACTTCACCGACACCGGCGTGGTGACCAAGTTC
CTGTTCAACGTGTACCTGGGCACCGTGCTGAGCCACTACTACGTGATGCC
CCTGACCTGCAACAGCGCCCTGAAGCTGGAGTACTGGGTGACCCCCCTGA
CCAGCAAGCAGTACCTGCTGGCCTTCAACCAGGACGGCGTGATCTTCAAC
GCCGTGGACTGCAAGAGCGACTTCATGAGCGAGATCAAGTGCAAGACCC
TGAGCATCGCCCCCCTGACCGGCGTGTACGAGCTGAACGGCTACACCGTG
CAGCCCATCGCCGACGTGTACCGCCGCATCCCCACCCTGCCCGACTGCAA
CATCGAGGCCTGGCTGAACGACAAGAGCGTGCCCAGCCCCCTGAACTGG
GAGCGCAAGACCTTCAGCAACTGCAACTTCAACATGAGCAGCCTGATGA
GCTTCATCCAGGCCGACAGCTTCATCTGCAACAACATCGACGCCGCCAAG
ATCTACGGCATGTGCTTCAGCAGCATCACCATCGACAAGTTCGCCATCCC
CAACGGCCGCAAGGTGGACCTGCAGCTGGGCAACCTGGGCTACCTGCAG
AGCTTCAACTACCGCATCGACACCACCGCCACCAGCTGCCAGCTGTACTA
CAACCTGCCCGCCGCCAACGTGAGCGTGAGCCGCTTCAACCCCAGCACCT
GGAACCGCCGCTTCGGCTTCACCGAGCAGAGCGTGTTCAAGCCCCAGCCC
GCCGGCGTGTTCACCGACCACGACGTGGTGTACGCCCAGCACTGCTTCAA
GGCCCCCACCAACTTCTGCCCCTGCAAGCTGGACGGCAGCCTGTGCGTGG
GCAGCGGCAGCGGCATCGACGCCGGCTACAAGACCAGCGGCATCGGCAC
CTGCCCCGCCGGCACCAACTACCTGACCTGCTACAACGCCGCCCAGTGCG
ACTGCATGTGCACCCCCGACCCCATCACCAGCAAGGCCACCGGCCCCTAC
AAGTGCCCCCAGACCAAGTACCTGGTGGGCATCGGCGAGCACTGCAGCG
GCCTGGCCATCAAGAGCGACCACTGCGGCGGCAACCCCTGCACCTGCCAG
CCCCAGGCCTTCCTGGGCTGGAGCGTGGACAGCTGCCTGCAGGGCGACCG
CTGCAACATCTTCGCCAACTTCATCCTGCACGGCGTGAACAGCGGCACCA
CCTGCAGCACCGACCTGCAGAAGAGCAACACCGACATCATCCTGGGCGT
GTGCGTGAACTACGACCTGTACGGCATCACCGGCCAGGGCATCTTCGTGG
AGGTGAACGCCACCTACTACAACAGCTGGCAGAACCTGCTGTACGACAG
CAACGGCAACCTGTACGGCTTCCGCGACTACCTGACCAACCGCACCTTCA
TGATCCGCAGCTGCTACAGCGGCCGCGTGAGCGCCGCCTTCCACGCCAAC
AGCAGCGAGCCCGCCCTGCTGTTCCGCAACATCAAGTGCAACTACGTGTT

CAACAACACCCTGAGCCGCCAGCTGCAGCCCATCAACTACTTCGACAGCT
ACCTGGGCTGCGTGGTGAACGCCGACAACAGCACCAGCAGCGTGGTGCA
GACCTGCGACCTGACCGTGGGCAGCGGCTACTGCGTGGACTACAGCACCA
AGCGCCGCAGCCGCGGCGCCATCACCACCGGCTACCGCTTCACCAACTTC
GAGCCCTTCACCGTGAAGAGCGTGAACGACAGCCTGGAGCCCGTGGGCG
GCCTGTACGAGATCCAGATCCCCAGCGACTTCACCATCGGCAACATGGAG
GAGTTCATCCAGACCAGCAGCCCCAAGGTGACCATCGACTGCAGCACCTT
CGTGTGCGGCGACTACGCCGCCTGCAAGAGCCAGCTGGTGGAGTACGGC
AGCTTCTGCGACAACATCAACGCCATCCTGACCGAGGTGAACGAGCTGCT
GGACACCACCCAGCTGCAGGTGGCCAACAGCCTGATGAACGGCGTGACC
CTGAGCACCAAGCTGAAGGACGGCGTGAAGTTCAACGTGGACGACATCA
ACTTCAGCCCCGTGCTGGGCTGCGTGGGCAGCGACTGCAACCGCGTGAGC
AGCCGCAGCGCCATCGAGGACCTGCTGTTCAGCAAGGTGAAGCTGGCCG
ACGTGGGCTTCGTGGAGGCCTACAACAACTGCACCGGCGGCGCCGAGAT
CCGCGACCTGATCTGCGTGCAGAGCTACAACGGCATCAAGGTGCTGCCCC
CCCTGCTGAGCGAGAACCAGATCAGCGGCTACACCCTGGCCGCCACCAGC
GCCAGCCTGTTCCCCCCCTGGAGCGCCGCCGCCGGCGTGCCCTTCTACCT
GAACGTGCAGTACCGCATCAACGGCATCGGCGTGACCATGGACGTGCTG
AGCCAGAACCAGAAGCTGATCGCCAACGCCTTCAACAACGCCCTGGGCG
CCATCCAGGAGGGCTTCGACGCCACCAACAGCGCCCTGGTGAAGATCCA
GGCCGTGGTGAACGCCAACGCCGAGGCCCTGAACAACCTGCTGCAGCAG
CTGAGCAACCGCTTCGGCGCCATCAGCAGCAGCCTGCAGGAGATCCTGAG
CCGCCTGGACGCCCTGGAGGCCCAGGCCCAGATCGACCGCCTGATCAACG
GCCGCCTGACCGCCCTGAACGCCTACGTGAGCCAGCAGCTGAGCGACAG
CACCCTGGTGAAGTTCAGCGCCGCCCAGGCCATGGAGAAGGTGAACGAG
TGCGTGAAGAGCCAGAGCAGCCGCATCAACTTCTGCGGCAACGGCAACC
ACATCATCAGCCTGGTGCAGAACGCCCCCTACGGCCTGTACTTCATCCAC
TTCAGCTACGTGCCCACCAAGTACGTGACCGCCAAGGTGAGCCCCGGCCT
GTGCATCGCCGGCGACCGCGGCATCGCCCCCAAGAGCGGCTACTTCGTGA
ACGTGAACAACACCTGGATGTTCACCGGCAGCGGCTACTACTACCCCGAG
CCCATCACCGGCAACAACGTGGTGGTGATGAGCACCTGCGCCGTGAACTA
CACCAAGGCCCCCGACGTGATGCTGAACATCAGCACCCCCAACCTGCCCG
ACTTCAAGGAGGAGCTGGACCAGTGGTTCAAGAACCACACCAGCGTGGC
CCCCAACCTGAGCCTGGACTACATCAACGTGACCTTCCTGGACCTGCAGG
ACGAGATGAACCGCCTGCAGGAGGCCATCAAGGTGCTGAACCAGAGCTA
CATCAACCTGAAGGACATCGGCACCTACGAGTACTACGTGAAGTGGCCCT
GGTACGTGTGGCTGCTGATCGGCTTCGCCGGCGTGGCCATGCTGGTGCTG
CTGTTCTTCATCTGCTGCTGCACCGGCTGCGGCACCAGCTGCTTCAAGAA
GTGCGGCGGCTACCCCTACGACGTGCCCGACTACGCCTAA

Translated ORF

MFLILLISLPTAFAVIGDLKCTTVSINDVDTGVPSISTDTVDVTNGLGTYYVLD
RVYLNTTLLLNGYYPTSGSTYRNMALKGTLLLSTLWFKPPFLSDFTNGIFAK
VKNTKVNKKGVMYSEFPAITIGSTFVNTSYSVVVQPYTTNFDNKIQGLLEVS
VCQYTMCEYPNTICNPNLGNKRVELWHWDTGVVSCLYKRNFTYDVNADYL
YFHFYQEGGTFYAYFTDTGVVTKFLFNVYLGTVLSHYYVMPLTCNSALKLE
YWVTPLTSKQYLLAFNQDGVIFNAVDCKSDFMSEIKCKTLSIAPLTGVYELN
GYTVQPIADVYRRIPTLPDCNIEAWLNDKSVPSPLNWERKTFSNCNFNMSSL
MSFIQADSFICNNIDAAKIYGMCFSSITIDKFAIPNGRKVDLQLGNLGYLQSFN
YRIDTTATSCQLYYNLPAANVSVSRFNPSTWNRRFGFTEQSVFKPQPAGVFT
DHDVVYAQHCFKAPTNFCPCKLDGSLCVGSGSGIDAGYKTSGIGTCPAGTNY
LTCYNAAQCDCMCTPDPITSKATGPYKCPQTKYLVGIGEHCSGLAIKSDHCG
GNPCTCQPQAFLGWSVDSCLQGDRCNIFANFILHGVNSGTTCSTDLQKSNTDI
ILGVCVNYDLYGITGQGIFVEVNATYYNSWQNLLYDSNGNLYGFRDYLTNR
TFMIRSCYSGRVSAAFHANSSEPALLFRNIKCNYVFNNTLSRQLQPINYFDSY
LGCVVNADNSTSSVVQTCDLTVGSGYCVDYSTKRRSRGAITTGYRFTNFEPF
TVKSVNDSLEPVGGLYEIQIPSDFTIGNMEEFIQTSSPKVTIDCSTFVCGDYAA
CKSQLVEYGSFCDNINAILTEVNELLDTTQLQVANSLMNGVTLSTKLKDGVK
FNVDDINFSPVLGCVGSDCNRVSSRSAIEDLLFSKVKLADVGFVEAYNNCTG
GAEIRDLICVQSYNGIKVLPPLLSENQISGYTLAATSASLFPPWSAAAGVPFYL
NVQYRINGIGVTMDVLSQNQKLIANAFNNALGAIQEGFDATNSALVKIQAVV
NANAEALNNLLQQLSNRFGAISSSLQEILSRLDALEAQAQIDRLINGRLTALN
AYVSQQLSDSTLVKFSAAQAMEKVNECVKSQSSRINFCGNGNHIISLVQNAP
YGLYFIHFSYVPTKYVTAKVSPGLCIAGDRGIAPKSGYFVNVNNTWMFTGSG
YYYPEPITGNNVVVMSTCAVNYTKAPDVMLNISTPNLPDFKEELDQWFKNH
TSVAPNLSLDYINVTFLDLQDEMNRLQEAIKVLNQSYINLKDIGTYEYYVKW
PWYVWLLIGFAGVAMLVLLFFICCCTGCGTSCFKKCGGYPYDVPDYA-
